# Ultrasound in the differential diagnosis of inguinoscrotal pain

**DOI:** 10.1590/0100-3984.2018.51.3e2

**Published:** 2018

**Authors:** Maria Cristina Chammas

**Affiliations:** 1 Director of the Ultrasound Department of the Instituto de Radiologia do Hospital das Clínicas da Faculdade de Medicina da Universidade de São Paulo InRad/HC-FMUSP), Professor for the FMUSP Graduate Program in Radiology, Vice-President 2 of the World Federation for Ultrasound in Medicine and Biology (2017-2019). E-mail: mcchammas@hotmail.com.

The causes of abdominal pain are extremely varied^(^^1^^-^^4^^)^. Pain in the inguinoscrotal region had several differential diagnoses and can be acute or chronic in nature. Various conditions provoke intense pain and are potentially serious. The imaging examinations used in diagnosing the causes of such pain include X-ray, computed tomography, and ultrasound.

Because it is widely available and safe, with an excellent cost-benefit ratio, ultrasound is usually the first imaging study used after the clinical examination, in order to elucidate or confirm the diagnosis of a palpated lesion-often identified by the patient, who is in pain and requested the consultation-as well as to inform decisions regarding the treatment strategy (clinical or surgical). In cases of suspected hernia in the region, the main objective of an ultrasound examination is to confirm its presence, to distinguish among the types of hernias (e.g., inguinoscrotal and femoral) and to make the differential diagnosis with torsions in the scrotum, inflammation, infection, thrombosis, and trauma.

In clinical practice, inguinal hernias are the most common, accounting for approximately 75% of all abdominal wall hernias, are not always easily diagnosed, and can manifest with or without bulging on clinical inspection. In addition, the clinical diagnosis can be impeded by patient-related factors, such as being in immediate postoperative period, obesity, etc.^(^^5^^)^. When the presence of a hernia is confirmed, it is important to identify the contents of the hernia sac, as well as to determine whether the hernia is acute or chronic, as well as whether it is complicated^(^^6^^)^.

One complication of a hernia is incarceration, which occurs when the opening through which the hernia sac passes is wide but the sac can not be reduced. In such cases, Doppler mapping is fundamental, allowing vascularization to be identified in the herniated fat layer and in the wall of the herniated intestinal loop. When the hernia passes through a narrow opening in the abdominal wall and is voluminous, it can be complicated by strangulation. A strangulated hernia constitutes an emergency because the hernia is irreducible and the ischemia can progress to necrosis. Contrary to what is seen in an incarcerated hernia, Doppler mapping of a strangulated hernia shows no blood flow in the hernia sac, underscoring the emergency nature of the diagnosis^(^^5^^)^.

To make the correct diagnosis, it is necessary to know the anatomy of the region, as it appears on ultrasound, and its reference points; the proper examination technique (and equipment settings); and the appropriate dynamic maneuvers and patient positioning to be used during the examination. It is important to have a systematized protocol for ultrasound examination so that no steps are forgotten or neglected. In general, the ultrasound approach is sufficient and there is no need for additional imaging examinations. It is fundamental that the ultrasound technician be experienced. The characteristics observed in B-mode and (color or spectral) Doppler ultrasound examinations should be reported in an assertive manner, thus aiding the prescribing physician in the decision-making process and increasing the chances of therapeutic success.

A pictorial essay and review, published in this issue of Radiologia Brasileira^(^^7^^)^, focuses on the daily practice of physicians who perform ultrasound examinations of the inguinoscrotal region. The authors explore the examination technique, the anatomy of the region, and the major differential diagnoses of inguinoscrotal pain, as well as describing a number of cases, some common and others more rare. Certainly, physicians who work in the field of ultrasound, especially in emergency care, will benefit from reading the article cited.
